# The *Aeromonas caviae AHA0618* gene modulates cell length and influences swimming and swarming motility

**DOI:** 10.1002/mbo3.233

**Published:** 2014-12-17

**Authors:** Rebecca C Lowry, Jennifer L Parker, Ramhari Kumbhar, Stephane Mesnage, Jonathan G Shaw, Graham P Stafford

**Affiliations:** 1 Department of Infection and Immunity, University of Sheffield Medical SchoolBeech Hill Road, Sheffield, S10 2RX, UK; 2 School of Clinical Dentistry, Claremont Crescent, University of SheffieldSheffield, S10 2TA, UK; 3 Department of Molecular Biology and Biotechnology, Firth Court, Western BankSheffield, S10 2TN, UK

**Keywords:** *Aeromonas*, flagella, morphology, motility, peptidoglycan

## Abstract

*Aeromonas caviae* is motile via a polar flagellum in liquid culture, with a lateral flagella system used for swarming on solid surfaces. The polar flagellum also has a role in cellular adherence and biofilm formation. The two subunits of the polar flagellum, FlaA and FlaB, are posttranslationally modified by O-linked glycosylation with pseudaminic acid on 6–8 serine and threonine residues within the central region of these proteins. This modification is essential for the formation of the flagellum. *Aeromonas caviae* possesses the simplest set of genes required for bacterial glycosylation currently known, with the putative glycosyltransferase, Maf1, being described recently. Here, we investigated the role of the *AHA0618* gene, which shares homology (37% at the amino acid level) with the central region of a putative deglycosylation enzyme (HP0518) from the human pathogen *Helicobacter pylori*, which also glycosylates its flagellin and is proposed to be part of a flagellin deglycosylation pathway. Phenotypic analysis of an *AHA0618 A. caviae* mutant revealed increased swimming and swarming motility compared to the wild-type strain but without any detectable effects on the glycosylation status of the polar flagellins when analyzed by western blot analysis or mass spectroscopy. Bioinformatic analysis of the protein AHA0618, demonstrated homology to a family of l,d-transpeptidases involved in cell wall biology and peptidoglycan cross-linking (YkuD-like). Scanning electron microscopy (SEM) and fluorescence microscopy analysis of the wild-type and *AHA0618-*mutant *A. caviae* strains revealed the mutant to be subtly but significantly shorter than wild-type cells; a phenomenon that could be recovered when either *AHA0618* or *H. pylori HP0518* were introduced. We can therefore conclude that AHA0618 does not affect *A. caviae* behavior by altering polar flagellin glycosylation levels but is likely to have a role in peptidoglycan processing at the bacterial cell wall, consequently altering cell length and hence influencing motility.

## Introduction

The bacterial cell envelope is often enhanced by structures that contribute to pathogen virulence, such as flagella, fimbriae, and adhesins. Changes in the number or characteristics of any of these cell surface appendages can lead to altered bacterial behavior, such as attenuation or increased virulence of pathogenic strains.

The bacterial flagellum is one appendage that is considered to be an important virulence factor for several pathogenic microorganisms. This complex nanomachine not only permits the motility of a wide range of bacteria but also contributes to colonization of human niches and in some cases adhesion to target cells (Eaton et al. 1996; Pratt and Kolter 1998; Rabaan et al. 2001; Giron et al. 2002; Tomich et al. 2002). Bacterial flagella are composed of a helical filament made up of repeating subunits of flagellin monomers that extend from the cell surface and are attached, via the hook, to a rotating basal body spanning the cell envelope (Chevance and Hughes 2008). It is assembled by means of a dedicated Type III secretion system that transports flagella structural proteins through a central lumen before assembly at the distal tip (Evans et al. 2014).

In recent years, it has been established that a number of bacteria can posttranslationally modify their flagellin protein subunits by the addition of sugars via the *O*-glycosylation of serine or threonine residues (Zunk and Kiefel 2014). These include pathogens such as *Aeromonas* (Tabei et al. 2009), *Helicobacter* (Josenhans et al. 2002; Schirm et al. 2003), and *Campylobacter* (Thibault et al. 2001), all of which glycosylate their flagella with nine carbon sugars (nonulosonic acids) of the sialic acid family such as pseudaminic or legionaminic acid (Logan 2006; Nothaft and Szymanski 2010; Zunk and Kiefel 2014). This modification is considered essential for the formation of the flagellum and therefore influences virulence in these pathogenic species (Josenhans et al. 2002; Goon et al. 2003; Schirm et al. 2003; Tabei et al. 2009; Wilhelms et al. 2012; Parker et al. 2014). However, the reasons why some pathogenic bacteria glycosylate their flagella and why others, such as *Escherichia coli* and *Salmonella* spp., do not, is currently unknown.

This study focuses on *Aeromonas caviae*, Sch3, a strain that possesses the smallest set of glycosylation genes currently known for pseudaminic acid biosynthesis and its transfer onto flagellins (Tabei et al. 2009; Parker et al. 2012, 2014). *Aeromonas caviae* is therefore an ideal model organism for the study of bacterial glycosylation when compared to the 20–50 genes found in the glycosylation islands of *Campylobacter* species. *Aeromonas* species are the cause of wound and enteric infections in humans (Parker and Shaw 2011) and their flagella are important colonization factors. *Aeromonas caviae* Sch3 is motile in liquid environments through the use of a single polar flagellum composed of two flagellin proteins, FlaA and FlaB, that are *O*-glycosylated with 6–8 pseudaminic acid (Pse5Ac7Ac) residues (Rabaan et al. 2001; Tabei et al. 2009). These Pse5Ac7Ac residues are transferred onto flagellins in the cytoplasm before their secretion through the flagellar type III secretion system and polymerization into mature filaments (Parker et al. 2014). It is likely that the enzymes responsible for this modification are a novel family of putative glycosyltransferases, the Maf (motility-associated factor) proteins, which are encoded by genes located within flagellar assembly or glycosylation islands in a range of bacteria (Karlyshev et al. 2002; Schirm et al. 2003; Canals et al. 2007; Parker et al. 2012). *Aeromonas caviae* Sch3 possesses only one Maf protein, whereas some strains of *Campylobacter* have seven *maf* genes whose expression can be phase variable, potentially allowing *Campylobacter* to decorate its flagellins with a variety of pseudaminic acid or legionaminic acid derivatives during different stages of infection (Karlyshev et al. 2002; van Alphen et al. 2008). In addition, mutational studies carried out by Howard et al. (2009) demonstrated that altering the glycosylation of the *Campylobacter* flagellum changes its surface charge, subsequently adjusting bacterial behavior (such as ability to form biofilms and autoagglutinate).

In addition to the activity of specific glycosyltransferases, studies in *Helicobacter pylori* lead to the hypothesis that the levels or pattern of glycosylation is modulated by a possible glycosylation/deglycosylation homeostasis pathway which might act via a putative deglycosylation enzyme, HP0518 (Asakura et al. 2010). A *H. pylori HP0518* mutant was shown to be hypermotile and had “superior colonization capabilities” (Asakura et al. 2010). Furthermore, the *H. pylori HP0518* mutant demonstrated greater amounts of flagellin (FlaA) glycosylation and mass spectrometry demonstrated increased levels of pseudaminic acid on the flagellin (Asakura et al. 2010). However, recent reports from Sycuro et al. (2013) have indicated that HP0518 (which they named Csd6) is also involved in peptidoglycan processing at the cell surface and their *HP0518* mutant displayed altered cell shape (a “straight rod” phenotype). Additionally, purified recombinant HP0518 (Csd6) demonstrated l,d-carboxypeptidase activity (Sycuro et al. 2013). In light of these studies, we set out to examine the function of a putative HP0518 homolog, named AHA0618, identified from an in-house annotation of the unpublished *A. caviae* Sch3 draft genome sequence (J. G. Shaw, unpubl. data). Our aim was to investigate whether the AHA0618 gene product might be involved in a possible deglycosylation step in the *A. caviae* flagellin glycosylation pathway that acts to modulate pseudaminic acid levels on its flagellin and regulate cellular behavior. Here, we show that mutation of *AHA0618* affects *A. caviae* swimming and swarming motility, but despite these behavioral changes, flagellin glycosylation levels do not appear to be altered in this bacterium.

## Materials and Methods

### Bacterial strains, plasmids, and growth conditions

Bacterial strains and plasmids used in this study are listed in Table1. *Escherichia coli* strains were grown in Luria–Bertani (LB) Miller broth and on LB Miller agar, while *Aeromonas* strains were grown in brain heart infusion broth (BHIB) or on Columbia blood agar (Oxoid, Basingstoke, UK). Growth of *E. coli* and *Aeromonas* strains was typically carried out at 37°C. Ampicillin (50 *μ*g/mL), nalidixic acid (50 *μ*g/mL), kanamycin (50 *μ*g/mL), gentamicin (25 *μ*g/mL), streptomycin (50 *μ*g/mL), and chloramphenicol (25 *μ*g/mL) were added when necessary.

**Table 1:** Strains and plasmids used in this study

Strain or plasmid	Genotype and use or description	Source or reference
*Escherichia coli* strains		
DH5*α*	F^−^ Phi80*dlacZ* ΔM15 Δ(*lacZYA*-*argF*)U169 *deoR recA1 endA1* *hsdR17*(rK-mK+) *phoA supE*44 lambda-*thi*-1; used for general cloning	Invitrogen
S17-1*λpir*	*hsdR pro recA*, RP4-2 in chromosome, Km::Tn*7* (Tc::Mu) *λpir*, Tp^r^ Sm^r^	de Lorenzo et al. (1990)
*CC118 λpir*	Δ(*ara leu*)*7697 araD139* Δ*lacX74 galE galK phoA20 thi-1 rspE rpoB*(Rf^r^) *argE*(Am) *recA1 λpir*^+^	Herrero et al. (1990)
*Aeromonas* strains		
*A. caviae* Sch3N	Sch, spontaneous Nal^r^	Gryllos et al. (2001)
*A. caviae* JPS04	Sch3N; *AHA0618*::km^r^	This work
Plasmids		
pGEMT-EASY	Cloning vector, Amp^r^	Promega
pUC4KIXX	Source of Tn5-derived *nptII* gene, Km^r^	Pharmacia
pKNG101	*ori*R6K *mob*RK2 *strAB sacBR*, 6.8 kb, Sm^r^	Kaniga et al. (1991)
pSRK(Gm)	pBBR1MCS-5-derived broad-host-range expression vector containing lac promoter and lacI^q^, lacZ*α*^+^, and Gm^r^	Khan et al. (2008)
pSRK_*AHA0618*	pSRK(Gm) containing *AHA0618* in Nde*I*/Bam*HI* site of MCS	This work
pSRK_*HP0518*	pSRK(Gm) containing *HP0518* in Nde*I*/Bam*HI* site of MCS	This work

### General DNA methods

Where required, DNA restriction endonucleases, T4 DNA ligase, and alkaline phosphatase were used as recommended by the suppliers (NEB, New England Biolabs (United Kingdom), Hitchin, UK).

### Generation of AHA0618 disruption mutant

The *AHA0618* disruption mutant was created by insertion of the Tn*5*-derived kanamycin resistance cartridge (*nptII*) from pUC4-KIXX (GE Healthcare Life Sciences, Little Chalfont, UK). For mutation of *AHA0618*, the gene was amplified using Expand High-Fidelity DNA polymerase (Roche, Burgess Hill, UK) with primers JLP_32 and JLP_33 to produce a DNA fragment of 0.4 kb, this was subsequently ligated into pGEMT-Easy. The 1.4-kb *SmaI*-digested kanamycin resistance cartridge from pUC4-KIXX was inserted into a *SmaI* restriction site in the middle of the *AHA0618* gene. The *AHA0618::km* construct was then ligated into the suicide vector pKNG101 (Kaniga et al. 1991) and transferred into *Aeromonas* by conjugation. Conjugal transfer of the recombinant plasmids from *E. coli* S17-1λ *pir* to *A. caviae* Sch3N was performed on Columbia blood agar for 6–8 h at 37°C. Serial dilutions of the mating mixture were then plated on LB agar supplemented with nalidixic acid and kanamycin; the latter antibiotic was added in order to select for recombination. Colonies that were kanamycin resistant (Km^r^) and streptomycin sensitive were selected for analysis by polymerase chain reaction (PCR) to confirm the mutation followed by phenotypic studies.

### Motility and swarming assays

To assess motility of *Aeromonas* strains, bacterial colonies were transferred with a sterile toothpick into the center of motility agar plates (1% tryptone, 0.5% NaCl, 0.25% agar). The plates were incubated face up at 25°C for 14–24 h, and motility was assessed by examining the migration of bacteria through the agar from the center toward the periphery of the plate.

To assess the swarming capabilities of *Aeromonas* strains, bacterial colonies were transferred with a sterile toothpick into the center of swarming agar plates (0.5% NaCl, 0.6% Difco Nutrient Broth, and 0.6% Eiken agar). The plates were incubated face up at 37°C for 16 h, and swarming was assessed by examining the migration of bacteria across the agar from the center toward the periphery of the plate (Kirov et al. 2004).

### Scanning electron microscopy

Scanning electron microscopy (SEM) samples were prepared by the University of Sheffield Electron Microscopy Unit (Department of Biomedical Sciences) in a gold sputter coater (Edwards S150b, Crawley, UK). Samples were analyzed on a Philips XL-20 SEM, Eindhoven, The Netherlands.

### Fluorescence microscopy

Fluorescence microscopy samples were prepared from overnight bacterial cultures in LB broth. Samples were allowed to dry on l-lysine-coated coverslips, fixed with paraformaldehyde, and then poststained with the thiol-reactive dye Alexa Fluor 594 (Life Technologies, Paisley, UK). Residual fluorescent dye was removed by washing cells with phosphate-buffered saline (PBS) and samples were mounted onto glass microscope slides with ProLong Antifade Gold with DAPI (Life Technologies). Samples were analyzed on a Zeiss Axiovert (Zeiss, Cambridge, UK) fluorescence microscope at 1000× magnification.

### Flagellin purification method

To purify *A. caviae* polar and lateral flagellins, a flagellar shearing method, adapted from Wilhelms et al. (2012) was carried out. *Aeromonas* strains were grown on large swarm agar plates (250 mL) overnight at 37°C. The resulting growth was scraped from the plates with PBS and flagella were sheared from the cells via the use of a blender for 10 min. Cells were removed from the suspension via centrifugation at 8000*g* for 30 min and debris removed from the supernatant by further centrifugation at 18,000*g* for 20 min. Flagella were pelleted via centrifugation at 75,000*g* for 1.5 h and resuspended in PBS.

### Lipopolysaccharide extraction and analysis

Lipopolysaccharide (LPS) was extracted from *Aeromonas* strains using an LPS extraction kit (ChemBio, St Albans, UK) according to the manufacturer's instructions. Briefly, cells from a 10 mL BHIB culture grown for 16 h were harvested and underwent lysis followed by incubation with chloroform. The supernatant was collected and the LPS purified via precipitation and wash steps. LPS samples were analyzed via Urea-SDS-PAGE (urea-sodium dodecyl sulfate-polyacrylamide gel electrophoresis) with a 12.5% resolving gel and analyses by silver staining (Guard-Petter et al. 1995).

### SDS-PAGE and immunoblotting

SDS-PAGE and immunoblotting of *Aeromonas* whole-cell preparations were carried out as described previously (Tabei et al. 2009). Briefly, *Aeromonas* strains were grown overnight in BHIB at 37°C. Equivalent numbers of cells were harvested by centrifugation. Cell pellets were boiled in SDS-PAGE loading buffer for 5 min. Protein samples were separated on SDS-PAGE gels (12% acrylamide). For immunoblotting, proteins were transferred onto a Hybond-C (GE Healthcare) nitrocellulose membrane. Following transfer, membranes were blocked with 5% (w/v) powdered skimmed milk. For identification of flagellin, membranes were probed with a polyclonal rabbit antipolar flagellin antibody (1:10,000) that only recognizes glycosylated flagellin or a rat antipolar flagellin antibody (1:1000) that recognizes both glycosylated and unglycosylated flagellin (Parker et al. 2014). A goat anti-rabbit or goat anti-rat horse radish peroxidase–conjugated secondary antibody (1:5000) was used in combination with the ECL detection system (GE Healthcare) before being exposed to X-ray film and developed using a Compact ×4 automatic film processor (Xograph Healthcare, Stonehouse, UK).

### Statistical analysis

The differences between the wild-type versus mutant strains and the mutant strains versus complemented strains were analyzed using GraphPad Prism 5.0 (GraphPad Software, Inc., La Jolla, CA). Data are given as mean ± standard error of the mean (SEM). Statistical significance was compared to the wild-type by *t*-test or one-way analysis of variance (ANOVA) (Tukey's multiple comparisons test).

### Bioinformatic tools

Protein BLAST searches were carried out using the NCBI BLAST tool (http://blast.ncbi.nlm.nih.gov/Blast.cgi) and conserve domain searches carried out with the NCBI conserved domain tool (http://www.ncbi.nlm.nih.gov/Structure/cdd/wrpsb.cgi) and the Pfam server (http://pfam.sanger.ac.uk/).

Manual protein alignments were performed using CLUSTALW (Thompson et al. 1994) (http://embnet.vital-it.ch/software/ClustalW.html) and displayed using box-shade (http://embnet.vital-it.ch/software/BOX_form.html).

Protein localization predictions were carried out using the Cello server (http://cello.life.nctu.edu.tw/).

### Accession number

*Aeromonas caviae* Sch3 AHA0618 accession number: HG934767.

## Results

### Bioinformatic analysis of *AHA0618* and the surrounding genome

**Figure 1:**
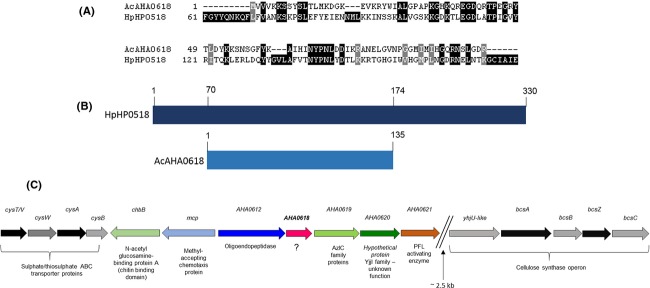
(A) Alignment of the deduced amino acid sequence of *Aeromonas caviae* Sch3N AHA0618 (accession HG934767) with the central region of homolog *Helicobacter pylori* 26695 HP0518 (amino acids 51–200) (Asakura et al. 2010). The alignment was performed using CLUSTALW (Thompson et al. 1994) and displayed using box shade. (B) Diagrammatic representation of the size of unfolded *A. caviae* Sch3 AHA0618 compared to *H. pylori 26695* HP0518. (C) Genetic organization of the genes surrounding *AHA0618* in the *A. caviae* Sch3 genome (J. G. Shaw, unpubl. data).

Analysis of the *A. caviae* Sch3 genome sequence (J. G. Shaw, unpubl. data) revealed the presence of a single *HP0518* homolog named *AHA0618*. The predicted amino acid sequence of *A. caviae* AHA0618 is 37% identical to the primary amino acid sequence level to the central region of the putative *H. pylori* deglycosylation enzyme HP0518 (Fig.1A and B). Using the Cello server (http://cello.life.nctu.edu.tw/) the predicted cellular localization of AHA0618 is cytoplasmic or periplasmic.

**Figure 2:**
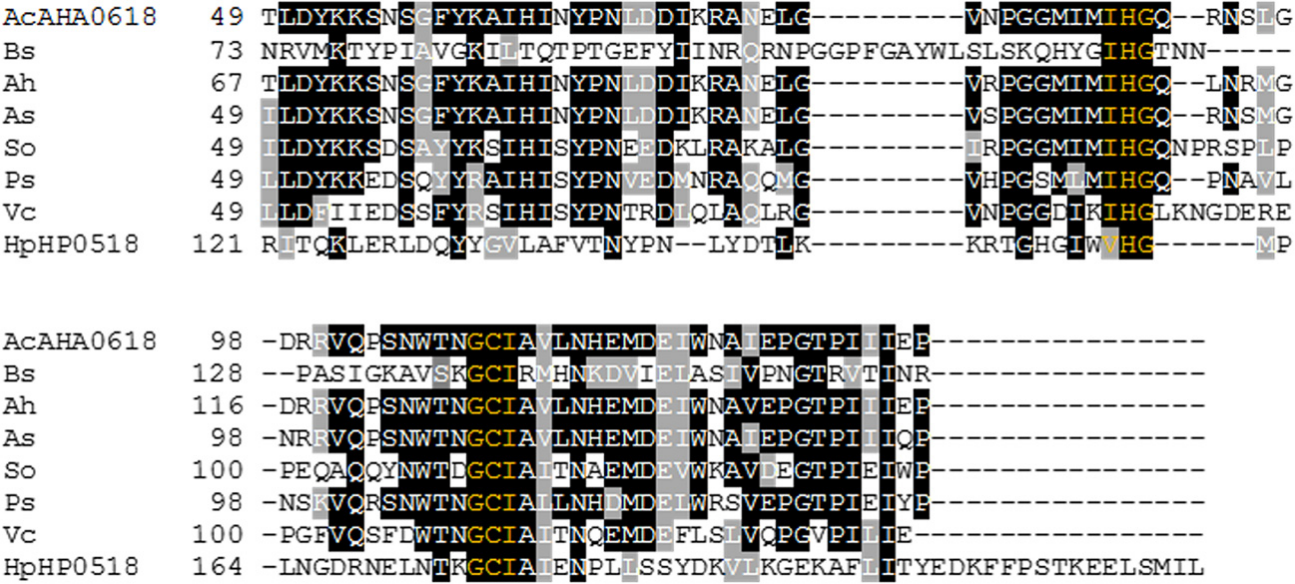
Alignment of *Aeromonas caviae* Sch3 *AHA0618* (AcAHA0618) and *Helicobacter pylori* 26695 *HP0518* (*HpHP0518*) with other members of the YkuD superfamily: *Bacillus subtilis* (Bs) (accession NP_389287), *Aeromonas hydrophila* (Ah) (accession AHE48181), *Aeromonas salmonicida* (As) (accession WP_021138855), *Shewanella oneidensis* (So) (accession NP_717783), *Plesiomonas shigelloides* (Ps) (accession WP_010864114), and *Vibrio cholera* (Vc) (accession NP_232564). The conserved histidine and cysteine residues from the his/cys motif in the predicted YkuD catalytic domain is highlighted.

When the primary amino acid sequence of AHA0618 was analyzed using both the NCBI conserved domain and Pfam prediction servers, it was found to be a member of the YkuD superfamily of proteins. This family of proteins contains a putative l,d-transpeptidase catalytic domain, present in a wide range of bacteria, often alongside peptidoglycan-binding domains, although this is not the case for *A. caviae*. YkuD, an l,d-transpeptidase, was originally characterized in *Bacillus subtilis* and has a highly conserved catalytic domain containing a histidine/cysteine motif invariant among members of this superfamily (Bielnicki et al. 2006). Its role is to influence the cell wall cross-linking of peptidoglycan and associated anchoring of the Lpp lipoprotein at the cell wall (Magnet et al. 2007), with work by Sanders and Pavelka (2013) demonstrating that *E. coli* lacking all of its YkuD homologs displays altered stress resistance. When aligned with a number of characterized and predicted members of this superfamily, AHA0618 and HP0518 were both shown to contain the conserved residues of the YkuD catalytic domain (Fig.2). Although many of the YkuD-type proteins shown in Figure2 do not contain the complete catalytic tetrad described in Bielnicki et al. (2006), the conserved histidine/cysteine residues are present in each. HP0518, however, like many of the other YkuD domain-containing proteins (e.g., YbiS, ErfK, YcfS of *E.coli*), is significantly larger than AHA0618. These data suggest a role for AHA0618 in the correct maintenance of the bacterial cell wall.

The genetic context of the *AHA0618* gene is intriguing since it is not associated with either of the two previously identified loci in *A. caviae* that contain all known genes required for flagellar filament assembly and its glycosylation (Rabaan et al. 2001; Tabei et al. 2009; Parker et al. 2012). Genes flanking *AHA0618* encode an oligoendopeptidase (M3 family) and an AzlC family protein which is potentially involved in amino acid transport and metabolism (Fig.1C). Homologs of the cellulose synthase operon from *Yersinia enterocolitica* (Fuchs et al. 2011), thought to be involved in exopolysaccharide production and genes encoding nutrient uptake apparatus (such as ABC transport systems), are also close by.

### *Aeromonas caviae AHA0618* mutant is hypermotile

To investigate the possible role of *AHA0618* in the *A. caviae* flagellin glycosylation pathway and to determine its true role, an insertion mutant was created. A kanamycin resistance cassette was inserted into the *AHA0618* gene in the same transcriptional orientation with the outward reading promoter in the cassette designed to reduce the occurrence of any polar effects downstream of the mutation. PCR using primers specific for *AHA0618* and the resistance cassette were used to assess whether construction of the mutant was successful (Table2). PCR confirmed both the location and orientation of the insertion mutation (data not shown).

**Table 2:** Primers used in this study

Primer name	Gene/use	Sequence 5′ to 3′ (restriction site)
T7 promoter	General sequencing of pGEM clones	TAATACGACTCACTATA
SP6	General sequencing of pGEM clones	ATTTAGGTGACACTATAG
Kan right	Mapping the location and orientation of the Kan cassette	TCATTTCGAACCCCAGAGTC
Kan left	Mapping the location and orientation of the Kan cassette	TGCTCCTGCCGAGAAAGTAT
JLP_32	*AHA0618* region for disruption – forward primer	(*Bam*HI) GGATCCCCTTGGCAGGGCCTCTGCATGG
JLP_33	*AHA0618* region for disruption – reverse primer	(*Bam*HI) GGATCCGGAAGGTGAAGCCATAGAGCAG
JLP_28	*A. caviae* Sch3N *AHA0618* for complementation and overexpression – forward primer	(*Nde*I) ATATATATCATATGGTGGTGGTGAAGAAGTC
JLP_29	*A. caviae* Sch3N *AHA0618* for complementation and overexpression – reverse primer	(*Bam*HI) TATTATGGATCCTCAGGGCTCGATGATGATGG
JLP_108	*H. pylori* 26695 *HP0518* for complementation and overexpression forward primer	(*Nde*I) CATATGAAAAAAATATTACCGGCTCTGTTAATG
JLP_109	*H. pylori* 26695 *HP0518* for complementation and overexpression – reverse primer GGATCCCTATTTTTCCATTATAATAGACACTTGATTGTT	(*Bam*HI)

**Figure 3:**
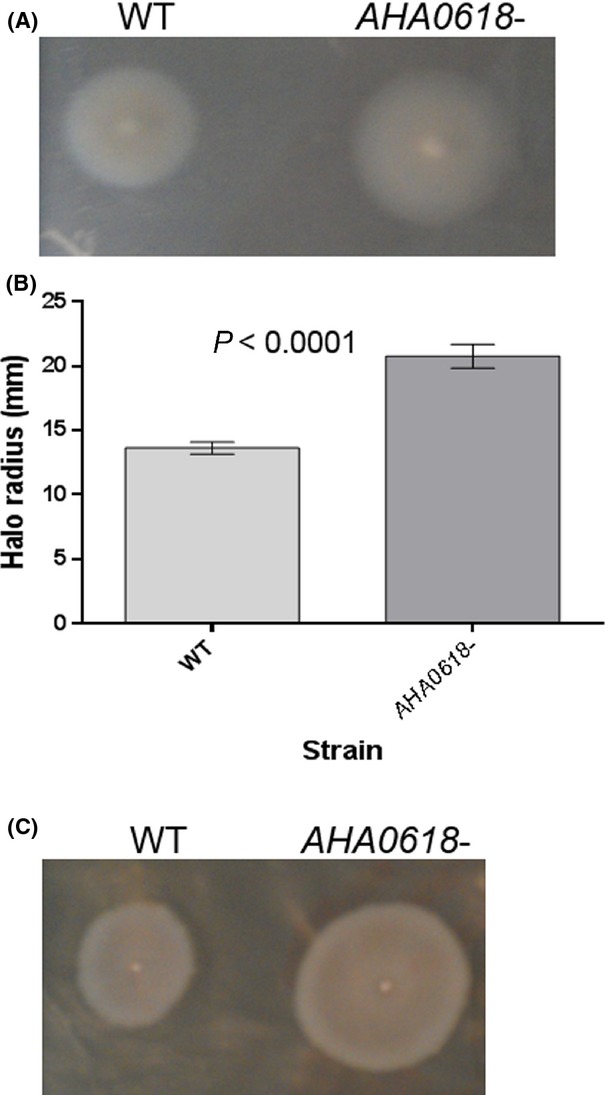
(A) Analysis of *Aeromonas caviae AHA0618*-mutant motility (*AHA0618*) compared to *A. caviae* Sch3N (wild type [WT]). Swimming motility assays were carried out on 0.25% semisolid agar. (B) The radius of each motility halo was measured and average measurements for the motility of *A. caviae* Sch3N (WT) and the *AHA0618* mutant (*AHA0618*) are presented here (*n* = 8) ± the standard error of the mean. A paired *t*-test carried out on the two datasets generated a *P* < 0.0001. (C) Analysis of *A. caviae AHA0618* mutant (*AHA0618*) swarming motility compared to *A. caviae* Sch3N (WT). Swarming assays were carried out on 0.6% swarming agar.

To test the effect of the *AHA0618* gene disruption on *A. caviae* motility, swimming motility assays were performed using semisolid agar (0.25% w/v) alongside the *A. caviae* wild-type strain (Sch3N). The *AHA0618* mutant displayed increased motility compared to the wild-type strain, with an average motility halo radius of 13.6 mm for the wild type, compared to 20.8 mm for the mutant, an increase in 1.5-fold (*P* < 0.0001) (Fig.3A and B). The *AHA0618* mutant was also found to be hypermotile on swarming agar when compared to the wild-type strain, although swarming motility could not be quantified appropriately due to irregular colony formation (Fig.3C). The hypermotility observed on swarming agar suggests that *AHA0618* may have a wider role than solely effecting polar flagella-mediated motility.

**Figure 4:**
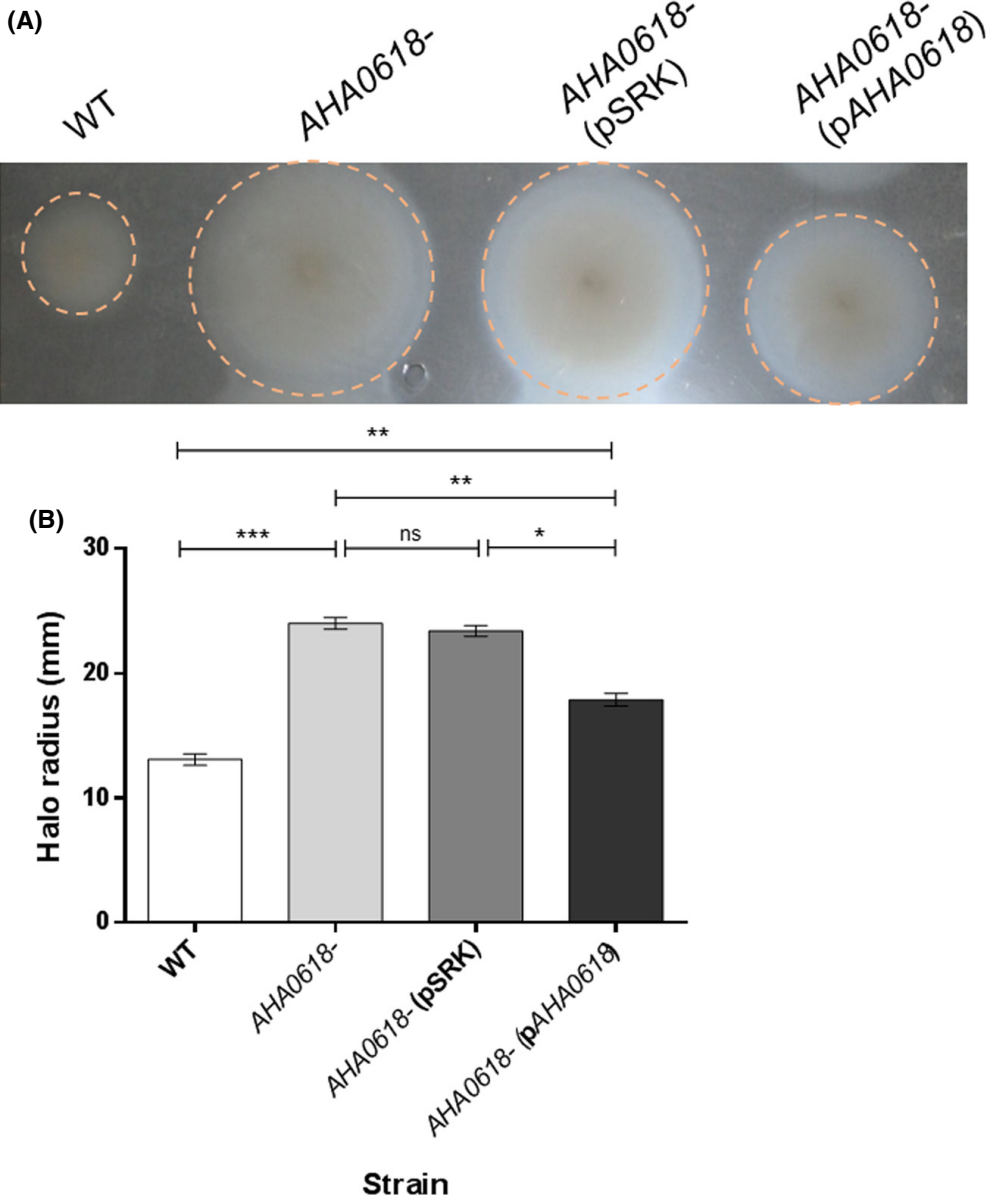
(A) Complementation analysis of pSRK_*AHA0618* in an *Aeromonas caviae AHA0618* mutant. Swimming motility assays were carried out on 0.25% semisolid agar for *A. caviae* Sch3N (WT), the *AHA0618* mutant (*AHA0618*), the mutant containing empty pSRK(Gm) (*AHA-*pSRK) and the mutant containing pSRK_*AHA0618* (*AHA0618* p*AHA0618*). (B) The radius of each motility halo was measured and average measurements are presented here (*n* = 5) ± the standard error of the mean. A one-way ANOVA, with a Tukey's multiple comparison test, was carried out on the datasets. ns, not significant; *P = 0.01–0.05; **P = 0.001–0.009; ***P = 0.0001–0.0009.

To test whether the *AHA0618* mutant could be complemented and thus discount any polar effects on the genes downstream, *AHA0618* was expressed in the IPTG-inducible vector, pSRK (Gm^r^) (Table1) and introduced into the mutant via conjugation. Motility was reduced to almost wild-type levels when the swimming motility of the complemented strain was assayed on semisolid agar (0.25% w/v) (Fig.4A) and this reduction in motility was found to be statistically significant (Fig.4B). Overexpression studies were also carried out where *AHA0618* was introduced into wild-type *A. caviae*; no differences in motility between the overexpression strain and wild-type *A. caviae* were detected indicating there was no negative dominance of this gene (Fig. S1).

### Glycosylation analysis of flagellin in *AHA0618* mutant and overexpression strains reveal no differences compared to the wild type

**Figure 5:**
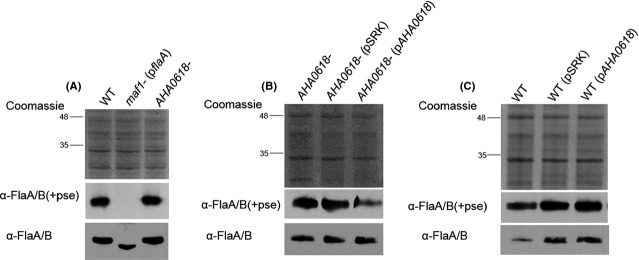
Western blot analysis of whole-cell protein preparations from *Aeromonas caviae* with a rabbit anti-polar flagellin antibody [*α*-FlaA/B(+pse)] (1:10,000) that recognizes only glycosylated flagellin and a rat antipolar flagellin antibody [*α*-FlaA/B] (1:1000) that recognizes both glycosylated and unglycosylated flagellin. (A) Lane 1, *A. caviae* Sch3N (WT); lane 2, *maf1* mutant containing pSRK_*flaA* (*maf1* p*flaA*); lane 3, *AHA0618* mutant (*AHA0618*). (B) Lane 1, *A. caviae AHA0618* mutant (*AHA0618*); lane 2, *AHA0618* mutant containing empty pSRK (*AHA0618*pSRK); lane 3, *AHA0618* mutant containing pSRK_*AHA0618* (*AHA0618*p*AHA0618*). (C) Lane 1, *A. caviae* Sch3N (WT); lane 2, Sch3N containing empty pSRK (WT pSRK); lane 3, Sch3N containing pSRK_*AHA0618* (WT p*AHA0618*). Whole-cell proteins were obtained from bacteria grown overnight at 37°C in brain heart infusion broth (BHIB).

Given the homology of AHA0618 to the HP0518 protein and their similar motility phenotypes, we hypothesized that AHA0618 could be having a similar role in *A. caviae* by potentially acting as a glycosylation modulator during the polar flagellin glycosylation pathway. We therefore investigated the potential presence of elevated levels of glycosylation on the *AHA0618-*mutant flagellins. To test whether *AHA0618* has an effect on the glycosylation status of the polar flagellins FlaA/B, western blot analysis was carried out on whole-cell samples of wild-type *A. caviae* and the *AHA0618* mutant (Fig.5A). Samples were probed with an anti-FlaA/B(+Pse) antibody that recognizes only the glycosylated polar flagellins (Tabei et al. 2009; Parker et al. 2012) and an anti-FlaA/B antibody that recognizes both glycosylated and unglycosylated flagellin (Parker et al. 2014). A *maf1* mutant (overexpressing the polar flagellin gene *flaA*) was used as a negative control when probed with the anti-FlaA/B(+Pse) antibody. The *maf1* gene encodes a putative pseudaminyltransferase responsible for transfer of activated pseudaminic acid on to flagellin monomers and the mutant, therefore only produces unglycosylated flagellins (Parker et al. 2012). Using our methods, unglycosylated FlaA/B cannot be detected in a *maf1-*mutant whole-cell preparation as the levels are too low, however, unglycosylated flagellin can be detected with an anti-FlaA/B antibody in a *maf1-*mutant strain where *flaA* is overexpressed from the multicopy plasmid pSRK. This reveals a decrease in size of the unglycosylated flagellins when compared to their glycosylated counterparts (Fig.5A). A size shift was not observed between wild-type and *AHA0618-*mutant flagellins when probing with either antibody; glycosylation is thus thought to be unchanged in these strains, despite the hypermotility seen in the *AHA0618* mutant. In addition, when mass spectrometry analysis of flagellins was carried out, identical glycopeptide patterns were observed for both the wild type and mutant; we therefore believe *AHA0618-*mutant flagellin glycosylation to be comparable to the wild type (data not shown).

Glycosylation status of the complemented *AHA0618*-mutant strain and the wild-type overexpressing *AHA0618* were also investigated by western blot analysis (Fig.5B and C, respectively); again the flagellins appeared at an identical size when compared to the original strains.

### *AHA0618* homolog, *H. pylori HP0518*, can complement the motility phenotype of *AHA0618 A. caviae* mutant

**Figure 6:**
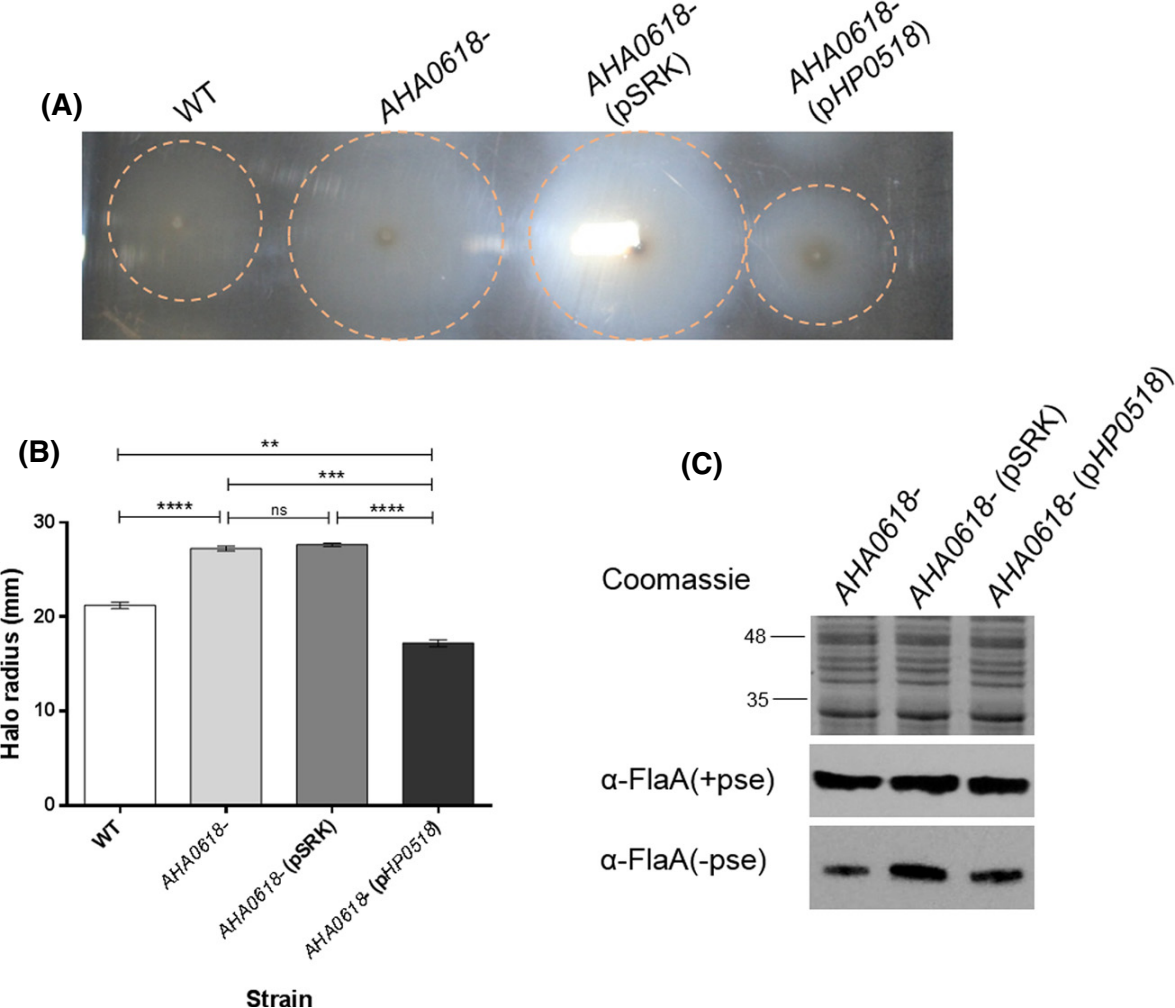
Analysis of pSRK_*HP0518* expression in an *Aeromonas caviae AHA0618* mutant. (A) Motility assays were carried out on 0.25% semisolid agar for *A. caviae* Sch3N (WT), the *AHA0618* mutant (*AHA0618*), the mutant containing empty pSRK(Gm) (*AHA0618*pSRK) and the mutant containing pSRK_*HP0518* (*AHA0618*p*HP0518*). (B) The radius of each motility halo was measured and average measurements are presented here (*n* = 5) ± the standard error of the mean. A one-way ANOVA, with a Tukey's multiple comparison test, was carried out on the datasets. ns = not significant; **P = 0.001–0.009; ***P = 0.0001–0.0009; ****P > 0.0001. (C) Western blot analysis of whole-cell protein preparations from *A. caviae* with a rabbit antipolar flagellin antibody [*α*-FlaA/B(+pse)] (1:10,000) that recognizes only glycosylated flagellin and a rat antipolar flagellin antibody [*α*-FlaA/B] (1:1000) that recognizes both glycosylated and unglycosylated flagellin. Lane 1, *A. caviae AHA0618* mutant (*AHA0618*); lane 2, *AHA0618* mutant containing empty pSRK(Gm) (*AHA0618*pSRK); lane 3, *AHA0618* mutant containing pSRK_*HP0518* (*AHA0618*p*HP0518*). Whole-cell proteins were obtained from bacteria grown overnight at 37°C in brain heart infusion broth (BHIB).

Since the related *HP0518* gene from *H. pylori* was shown to be a potential flagellin deglycosylase enzyme (Asakura et al. 2010), we tested the effect of expressing *HP0518* in *A. caviae* to see if this was the case when expressed in *A. caviae*, that is, did the extra domains in *HP0518* encode this function. *HP0518* from *H. pylori* 26695 was cloned into the IPTG-inducible vector pSRK (Gm), similar to *AHA0618*, and introduced into the *A. caviae AHA0618* mutant via conjugation. Analysis of swimming motility data from the resulting *A. caviae* strain revealed that *HP0518* was able to complement the increased-motility phenotype of the *A. caviae AHA0618* mutant and reduce motility to slightly below that of the wild-type strain (Fig6A and B). However, western blot analysis of whole-cell samples of the *AHA0618-*mutant strain expressing *HP0518* compared to the *AHA0618*-mutant alone, using the same antibodies as above, revealed no size differences between the flagellins (Fig.6C), indicating that, at least in *A. caviae*, these two genes do not affect flagellin glycosylation levels.

**Figure 7:**
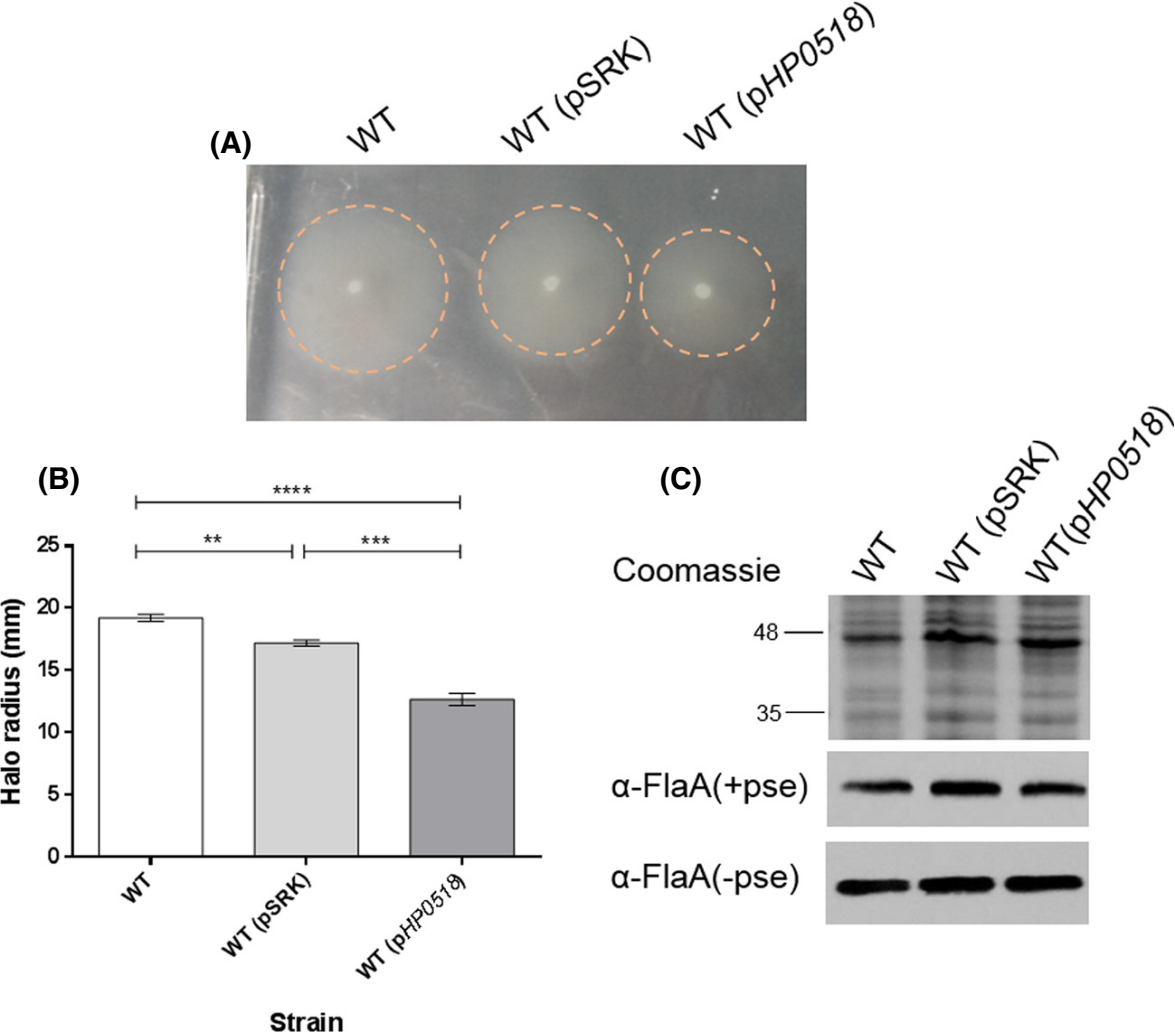
Analysis of pSRK_*HP0518* expression in an *Aeromonas caviae* Sch3N. (A) Motility assays were carried out on 0.25% semisolid agar for *A. caviae* Sch3N (WT), Sch3N containing empty pSRK(Gm) (WT pSRK) and Sch3N containing pSRK_*HP0518* (WT + *HP0518*). (B) The radius of each motility halo was measured and average measurements for the motility are presented here (*n* = 10) ± the standard error of the mean. A one-way ANOVA, with a Tukey's multiple comparison test, was carried out on the datasets. **P = 0.001–0.009; ***P = 0.0001–0.0009; ****P > 0.0001. (C) Western blot analysis of whole-cell protein preparations from *A. caviae* with a rabbit antipolar flagellin antibody [*α*-FlaA/B(+pse)] (1:10,000) that recognizes only glycosylated flagellin and a rat antipolar flagellin antibody [*α*-FlaA/B] (1:1000) that recognizes both glycosylated and unglycosylated flagellin. Lane 1, *A. caviae* Sch3N (WT); lane 2, Sch3N containing empty pSRK(Gm) (WT pSRK); lane 3, Sch3N containing pSRK_*HP0518* (Sch3N p*HP0518*). Whole-cell proteins were obtained from bacteria grown at 37°C in brain heart infusion broth (BHIB).

*HP0518* was also overexpressed in wild-type *A. caviae*. In contrast to the *A. caviae AHA0618* overexpression studies, motility assays revealed that *HP0518* overexpressed in the wild-type strain reduced its motility by 26.5% compared to the wild type containing the empty vector (*P* = 0.0001) (Fig.7A and B). Whole-cell samples of wild-type *A. caviae* expressing *HP0518* were then probed with both antibodies specific for FlaA/B and flagellins were compared to wild-type *A. caviae* samples. Again, no differences in flagellin size were observed (Fig.7C).

### Analysis of *A. caviae* AHA0618-mutant cellular envelope

In addition to its flagellins FlaA/B, *A. caviae* also modifies the LPS *O*-antigen with pseudaminic acid (Tabei et al. 2009). Mutations in genes of the pseudaminic acid biosynthetic pathway (*flmA*, *flmB*, *neuA*, *flmD*, *neuB*) lead to the loss of the sugar on the LPS *O*-antigen and therefore the LPS profiles of these mutants differ significantly to the wild type (Tabei et al. 2009; Parker et al. 2012). If AHA0618 was altering the LPS *O*-antigen glycosylation levels, this could lead to a change in bacterial cell surface charge and therefore effect how *A. caviae* interacts with its environment (and may help to explain why we see increased motility in the *AHA0618* mutant). LPS was extracted from wild-type and the *AHA0618*-mutant strains using a method described previously in Parker et al. (2012). However, the LPS profiles were found to be identical (Fig. S2), indicating that AHA0618 does not affect the levels of LPS *O*-antigen glycosylation.

### The *AHA0618* mutant displays altered cell length

**Figure 8:**
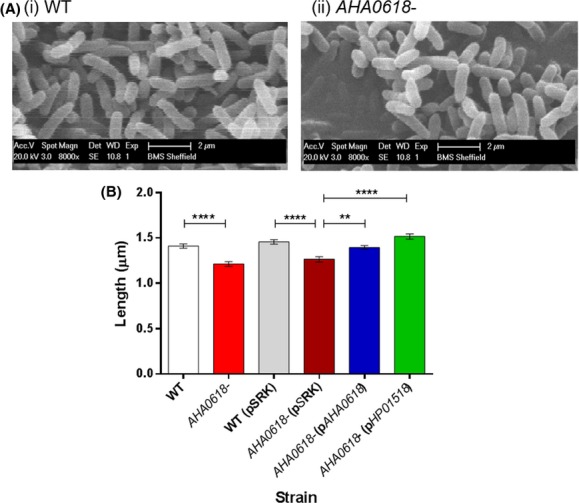
(A) Scanning electron microscopy of: (i) *Aeromonas caviae* Sch3N (WT) and (ii) *A. caviae AHA0618* mutant (*AHA0618-*), at 8000× magnification on a Philips XL-20 SEM. (B) Bacterial lengths were measured from micrographs of fluorescently labeled strains (see Materials and Methods section) of *A. caviae* Sch3N (WT), Sch3N containing empty pSRK(Gm) (WT pSRK), *AHA0618-*mutant *A. caviae* (*AHA0618-*), *AHA0618-*mutant *A. caviae* containing empty pSRK(Gm) (*AHA0618-*pSRK) and *AHA0618*-mutant *A. caviae* containing pSRK_*AHA0618* and pSRK_*HP0518* (*AHA0618-*pAHA0618 and *AHA0618-*p*HP0518*). Bacterial cell lengths were measured from four fields of view at 100× magnification and average cell lengths are presented here (*n* = 80) ± the standard error of the mean. A Tukey's multiple analysis test was carried out to assess the significance of the data. ****P < 0.0001, **P = 0.001–0.009.

A recent study by Sycuro et al. (2013) carried out cell morphology studies on *H. pylori* with mutations in genes encoding peptidoglycan modification enzymes. This investigation highlighted HP0518 (known as Csd6 in this study) as having l,d-carboxypeptidase activity and a mutant exhibited “straight rod morphology” as compared to the helical morphology of wild-type *Helicobacter*. Gross cell morphology of *A. caviae* wild-type and *AHA0618-*mutant strains was investigated by SEM, where no drastic change in cell shape was detected; however, a small difference in size between strains was observed (Fig.8A). This initial observation was supplemented using a higher throughput method of fluorescence microscopy of surface labeled cells (Alexa Fluor 594) and a subtle but significant difference in cell length was observed. In these studies, the *AHA0618 A. caviae* mutant displayed a 14% decrease in length compared to the mean of the wild type; these changes were proven to be statistically significant with a *t*-test when a total of 80 cell lengths were measured from four fields of view per strain during fluorescence microscopy (*P* < 0.0001) (Fig.8B).

**Figure 9:**
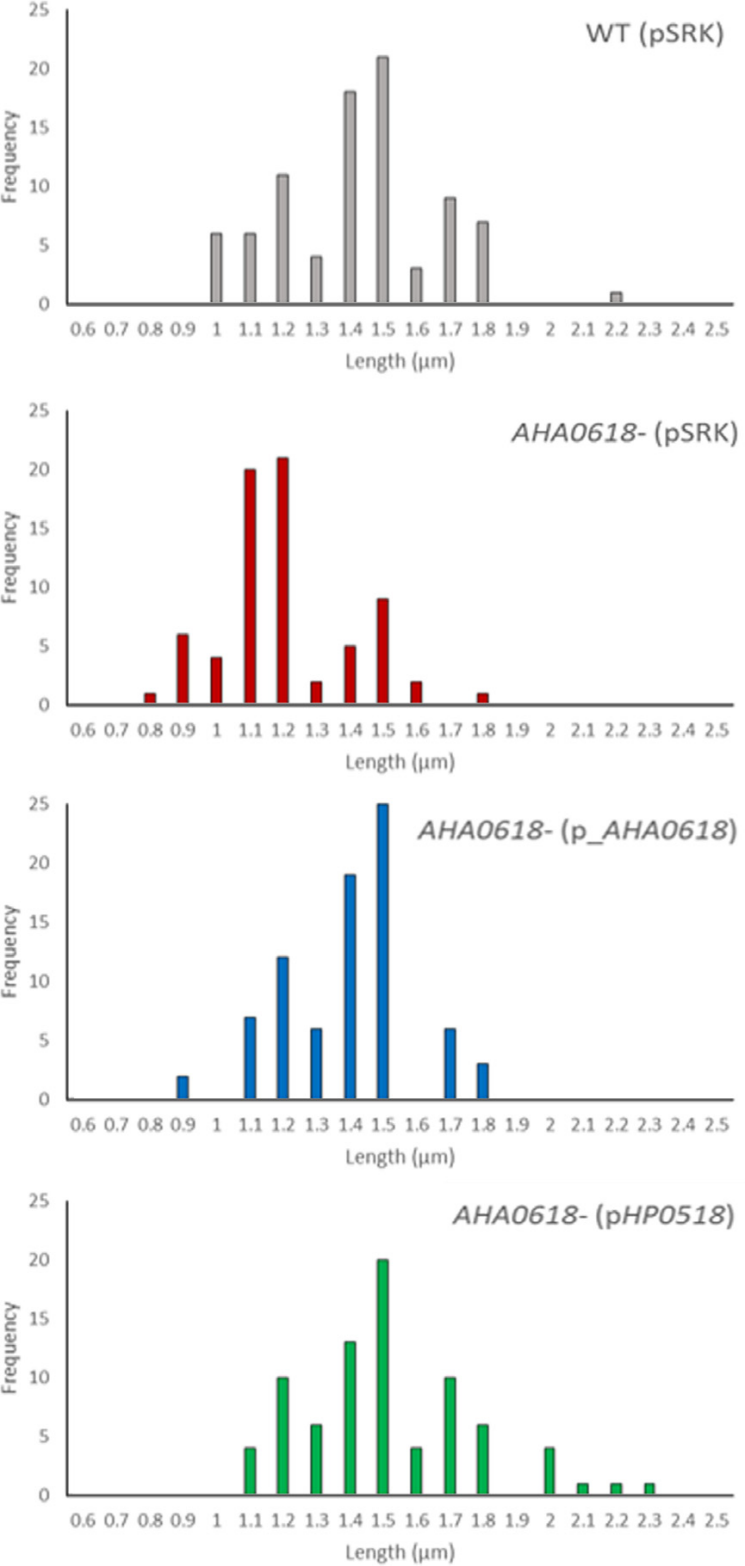
Frequency distribution of fluorescence microscopy cell length measurements (please refer to Fig.8B for details).

Additionally, when complemented with the *AHA0618* (pSRK_*AHA0618*) or *HP0518* (pSRK_*HP0518*) genes, the average cell length measurements of *AHA0618-*mutant strains showed a significant increase compared to *AHA0618* containing the empty vector (pSRK) (Fig.8B). As variations in average cell lengths were subtle, the frequency distribution of cell length measurements was analyzed between strains to see if the means masked details of the spread of cell length in these strains (Fig.9). The *AHA0618* mutant displayed the smallest minimum cell length of 0.77 *μ*m and was more frequently measured at the lower end of the frequency distribution scale, presenting a shift of the cell population length to the left (with a mode measurement of 1.2 *μ*m) when compared to the wild type (mode length of 1.5 *μ*m) (Fig.9). Frequency distribution of measurements taken from the *AHA0618* mutant containing either pSRK_*AHA0618* or pSRK_*HP0518* showed a shift to the right compared to the *AHA0618* mutant, displaying a similar pattern of cell lengths to the wild type, with both being most frequently measured at 1.5 *μ*m (Fig.9).

*Helicobacter pylori* naturally has a helical cell morphology, whereas *A. caviae* is a straight rod. Consequently, differences in cell morphology caused by *AHA0618* disruption may be far more subtle than a *csd6 H. pylori* disruption mutant. Here, cell length appears to be inversely correlated with observed bacterial motility of *A. caviae* strains analyzed.

Due to the differences in cell lengths observed, muropeptide analysis was carried out in triplicate on wild-type and *AHA0618*-mutant *A. caviae* peptidoglycan. These initial studies showed no differences in the muropeptide profiles between these two strains (data not shown).

## Discussion

In this study, we identified and examined the function of the *AHA0618* gene from *Aeromonas caviae*, a homolog of a *H. pylori* putative flagellin deglycosylation enzyme encoding gene, *HP0518*, that was hypothesized to participate in modulation of flagellin glycosylation in *H. pylori* (Asakura et al. 2010). A null mutant of the *A. caviae AHA0618* gene displays a hypermotile swimming and swarming phenotype similar to that of a *H. pylori HP0518* mutant, which suggested that AHA0618 may also be affecting flagellin glycosylation levels, adding a new level of complexity to this posttranslational modification pathway. Although the biological role of flagellin glycosylation is unknown, it is tempting to speculate that this modification could aid interactions with the bacterial environment and would potentially be beneficial if bacteria were capable of altering or at least maintaining the correct levels of glycosylation in response to environmental signals.

Despite an *AHA0618 A. caviae* mutant displaying a similar hypermotile phenotype to a *H. pylori HP0518* mutant, further analysis of the *A. caviae-*mutant flagellins suggests the glycosylation status to be indistinguishable from the wild type and that AHA0618 did not play a role in flagellin glycosylation in *A. caviae*. Furthermore, AHA0618 does not seem to affect the glycosylation status of the LPS *O*-antigen of *A. caviae*, indicating that the hypermotility of the mutant is unlikely to be due to altered cell surface characteristics from elevated glycosylation levels in the LPS.

To further examine the role of these genes from both organisms, we transplanted the *HP0518* gene into *A. caviae* and showed that it reduces the hypermotility phenotype in the same way as providing the AHA0618 gene in multicopy. However, in contrast to the *AHA0618* gene, heterologous expression of *HP0518* in wild-type *A. caviae* reduces motility, having a dominant negative effect. This suggests that while AHA0618 and HP0518 function similarly in *A. caviae*, there may be subtle differences in function possibly defined by the extra domains, of unknown function, contained within HP0518. Having discounted the mechanism of the motility phenotype being due to differences in glycosylation, we were drawn toward the recent work by Sycuro et al. (2013), indicating that *HP0518* (referred to as *csd6*) has l,d-carboxypeptidase activity and a *H. pylori cds6* mutant showed a drastic difference in cell morphology (straight rod in comparison to helical wild type). We therefore set out to examine if the AHA0618 could be functioning in a similar fashion, that is, in cell morphology due to peptidoglycan modification or cell wall architecture maintenance. This indeed appears to be the case since microscopy analysis of the *A. caviae AHA0618* mutant suggested a subtle difference in cell length compared the wild type and also demonstrated that the *AHA0618-*mutant strain expressing either *AHA0618* or *HP0518* displayed overall longer cell lengths compared to the mutant. Bioinformatics analysis which reveals homology of *AHA0618* with proteins shown to be involved in peptidoglycan cross-linking and lipopeptide anchoring at the bacterial cell wall (YkuD superfamily) (Bielnicki et al. 2006) and also possesses the conserved cysteine and histidine residues found in l,d-transpeptidases of the YkuD superfamily of proteins (along with the original YkuD characterized from *B. subtilis*) reinforcing that AHA0618 is likely to play a role in peptidoglycan modification. However, preliminary analysis of the muropeptide profiles of wild-type *A. caviae* and the *AHA0618* mutant found no variations between the strains, although very subtle differences can take much experimental optimization and complex further analysis. Furthermore, *AHA0618* seems to encode a much smaller protein than *H. pylori HP0518*, or other YkuD domain containing proteins from other bacteria, such as *E. coli*, possibly indicating differences in function that might explain the contradicting findings relating to this protein. For example, bifunctional penicillin-binding proteins have previously been described in a number of bacteria that contain transpeptidase and glycosyltransferase domains (reviewed in Sauvage et al. 2008); it is therefore possible that HP0518 has a dual role, possessing both a carboxypeptidase domain and a domain with deglycosylation activity. However, domain BLAST searches carried out with the HP0518 protein sequence did not identify any other putative functioning protein domains currently known.

The link between bacterial cell morphology and motility through various media has been recognized previously. The helical shape of *H. pylori* may help the gastric pathogen when in its specific niche, as straight rod mutants, such as a *H. pylori csd4* mutant (where *csd4* encodes a zinc metalloprotease with carboxypeptidase activity) displayed diminished directional motility compared to the naturally helical wild type through gel-like media (Sycuro et al. 2012, 2013). This mutant also displayed colonization defects in a mouse model (Sycuro et al. 2012). Studies in the Gram-positive bacterium, *Bacillus cereus*, also identified a putative cell wall peptidase, CwpFM; a mutant was found to have differences in cell morphology and motility compared to the wild-type strain (Tran et al. 2010). Mutant cells of this particular peptidase were found to be larger and less able to separate efficiently during cell division, creating bacterial chains. *Bacillus cereus cwpFM* mutants were therefore less motile than wild-type *B. cereus* (Tran et al. 2010) which further demonstrates that modifying peptidoglycan cross-linking at the cell wall can alter bacterial cell morphology, motility, and therefore bacterial behavior. Furthermore, recent work by Frirdich et al. (2014) described a peptidoglycan l,d-carboxypeptidase (Pgp2) that influences *Campylobacter jejuni* helical morphology, motility, and biofilm formation as when deleted, *C. jejuni* elicited straight rod cell morphology (similar to the *csd6/HP0518 H. pylori* strain; Sycuro et al. 2013).

One other phenotype displayed by peptidoglycan remodeling enzymes is their requirement to form gaps in the cell wall for the flagellar complex to assemble, although we have no evidence for this here (Scheurwater and Burrows 2011). Therefore, it is also possible that although we observe an effect on cell size, mutation of *AHA0618* may enhance the ability of the mutant flagella to form or rotate. For example, Roure et al. (2012) demonstrated that while *H. pylori* lacking MltD (a lytic transglycosylase) assemble flagella, they are nonmotile as they can no longer cleave the peptidoglycan backbone appropriately, resulting in flagella that are unable to rotate. The authors hypothesized this mutation may be affecting MotB peptidoglycan binding, potentially affecting torque generation (Roujeinikova 2008; Roure et al. 2012). However, deletions in peptidoglycan remodeling enzymes that enhance bacterial motility have not yet been reported and it is therefore likely that hypermotility of the *A. caviae* mutant seen here is due to bacterial size/morphology.

Taking all of our data, and that of others, into account, we seem to be have highlighted a link between bacterial size/morphology and velocity. This might be explained by differences in cell shape or length conferring changes in resistance (or drag) of the bacterial cell body moving through the aqueous environment that might thus alter the speed of swimming. In the case of *Aeromonas*, it is tempting to speculate that due to the physics of bacterial motility, larger cells move more slowly in solution and smaller cells more quickly with the wild-type cell length being tuned to its environment and modulated by auxiliary proteins such as AHA0618 to ensure optimal motility.

Although initial studies have shown no difference in muropeptide profiles between our two strains, our study demonstrates that the HP0518 homolog (Asakura et al. 2010); AHA0618 is not altering the glycosylation levels of *A. caviae* polar flagellins and is not effecting motility via these means. Therefore, our data support the role of these YkuD-family proteins in bacterial cell shape development where even the subtlest of changes to bacterial cell morphology may have an effect on bacterial motility and behavior.

## Conflict of Interest

None declared.

## Supporting Information

**Figure S1.** (A) Overexpression analysis of pSRK_*AHA0618* in an *Aeromonas caviae* Sch3N. Swimming motility assays were carried out on 0.25% semisolid agar for *A. caviae* Sch3N containing empty pSRK(Gm) (WT pSRK) and Sch3N containing pSRK_*AHA0618* (WT p*AHA0618*). Strains were not compared to *A. caviae* Sch3N due to the severe reduction in motility from addition of both empty and *AHA0618* containing plasmids. (B) The radius of each motility halo was measured and average measurements are presented here (*n* = 10) ± the standard deviation. A paired *t*-test comparing the two datasets generated a *P*-value of 0.214.

**Figure S2.** Analysis of lipopolysaccharide (LPS) isolated from *Aeromonas caviae* Sch3N (WT) and an *AHA0618* mutant (*AHA0618-*). LPS was extracted from bacteria grown at 37°C in BHIB, analyzed by SDS-PAGE (12%), and silver stained.
