# Supplementary figures and images for: The Aeromonas caviae AHA0618 gene modulates cell length and influences swimming and swarming motility
Source: Microbiologyopen. 2014 Dec 17;4(2):220–34. doi: 10.1002/mbo3.233 (PMC4398505; doi:10.1002/mbo3.233)

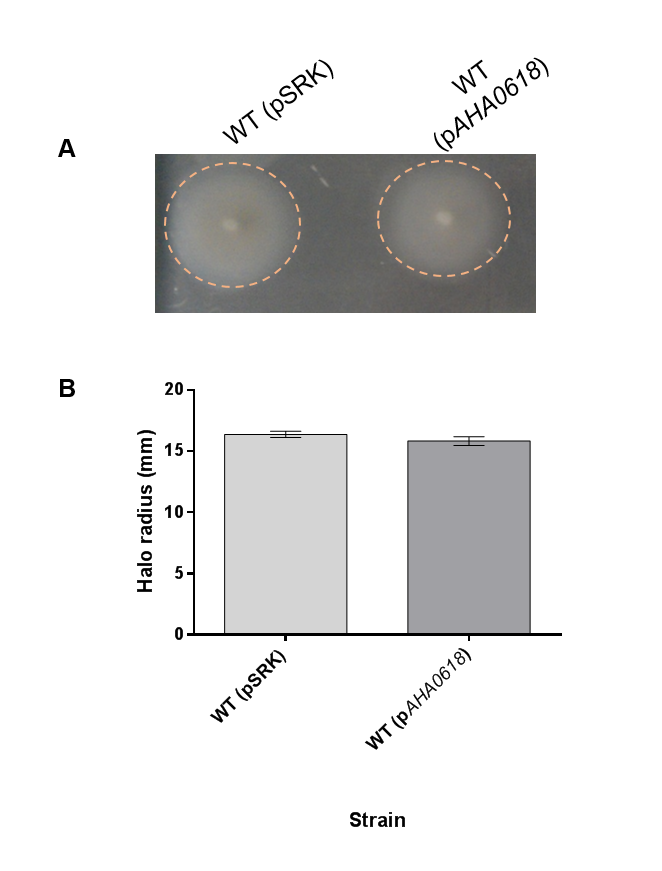

Supplement: Supplementary file 1 — Figure S1. (A) Overexpression analysis of pSRK_AHA0618 in an Aeromonas caviae Sch3N. Swimming motility assays were carried out on 0.25% semisolid agar for A. caviae Sch3N containing empty pSRK(Gm) (WT pSRK) and Sch3N containing pSRK_AHA0618 (WT pAHA0618). Strains were not compared to A. caviae Sch3N due to the severe reduction in motility from addition of both empty and AHA0618 containing plasmids. (B) The radius of each motility halo was measured and average measurements are presented here (n = 10) ± the standard deviation. A paired t-test comparing the two datasets generated a P-value of 0.214. [file mbo30004-0220-sd1.tif]

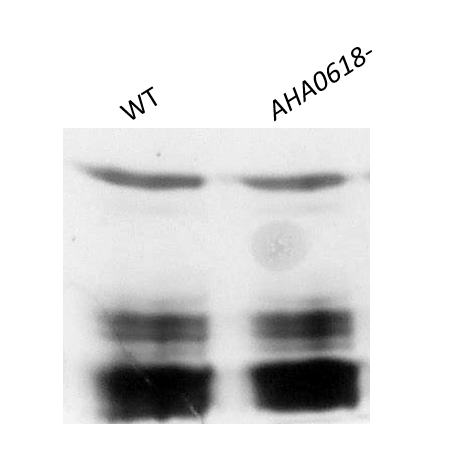

Supplement: Supplementary file 2 — Figure S2. Analysis of lipopolysaccharide (LPS) isolated from Aeromonas caviae Sch3N (WT) and an AHA0618 mutant (AHA0618-). LPS was extracted from bacteria grown at 37°C in BHIB, analyzed by SDS-PAGE (12%), and silver stained. [file mbo30004-0220-sd2.tif]
